# Medicine

**Published:** 1903-03

**Authors:** Theodore Fisher


### Progress of the Medical Sciences.

### MEDICINE.

**Abscess of the lung following pneumonia** forms the subject of two recent papers. The writers of both papers express their indebtedness for many of their facts to Eisendrath. The occurrence of abscess of the lung following pneumonia does not appear to be so uncommon as some physicians seem to think. Dr. Pye-Smith states in his article in Clifford Allbutt's *System of Medicine*, that he had never seen a case. Such experience must be very exceptional. Eisendrath succeeded in collecting no less than 96 recorded cases from literature. It may be mentioned, in passing, that I have met with five cases in the *post-mortem* room. In four of these cases it is true that the abscess was not large, but in one the whole of the right lower lobe had broken down into pus. It is an interesting fact, however, that this complication of pneumonia is not very fatal, and consequently only a minority of the cases are seen in the *post-mortem* room. Thus in the 25 recorded cases of acute simple abscess collected by Eisendrath, there were no deaths. All the abscesses, however, cannot be classified under the acute simple form. When the abscess is of the acute gangrenous type, the prognosis is more serious; but even then not so serious as might be expected. Of 28 recorded cases only six died. Where the abscesses become chronic, however, a fatal ending is more frequent.

The following is Eisendrath's clinical picture of abscess of the lung following pneumonia: Some short time after the crisis there is a rise of temperature, which becomes remittent. The sputum becomes purulent, and there is distressing cough associated with expectoration of pus in large quantities. Should, however, the abscess cavity not communicate with a bronchus, there is little expectoration. There is in all cases loss of appetite and a rapid decline in strength. Physical examination is often disappointing. The abscess is generally in one of the lower lobes; but there are no typical physical signs. Dulness is generally present; while the respiratory murmur, vocal resonance, and fremitus are usually diminished; but there may be bronchial breathing. The most reliable sign is the presence of large moist râles, not infrequently metallic in character. Another striking feature is the variability of the physical signs: at one time dulness, and then a tympanitic note at the same spot. H. A. Hare,[1] the joint author of one of the papers, records a case of abscess of the lung occurring after pneumonia, which was under his own care. A young woman, after nursing a case of croupous pneumonia, became affected with the same disease. After passing through a severe attack of pneumonia the temperature failed to return to normal, a "pseudo-crisis" taking place on the ninth day. After this the temperature gradually became of the septic type, and "sweats and chills" followed. There was a good deal of emaciation associated with persistent cough. Thirty days after the beginning of the attack of pneumonia, during a violent fit of coughing, the patient brought up "considerably over a teacupful of pus," which was not offensive, but nearly suffocated her. The dulness which had been present was supposed to have been due to delayed resolution. The expectoration of pus continued, so that it was decided, after a consultation with Prof. Da Costa, to operate. This was done, but the abscess cavity was not reached. Three days later, however, what appeared to have been a second abscess, containing

[1] *Med. News*, 1902, lxxxi. 385.

about half-a-pint of pus, suddenly ruptured, and again nearly produced death by suffocation. Although the patient was at this time in a very feeble condition, gradual improvement took place; the temperature became normal on the 73rd day of her illness, and she was discharged on the 103rd day. She eventually regained perfect health.

Dr. McRae[1] also records two cases of abscess of the lung following pneumonia, in both of which an operation was performed. The first patient, a youth aged 19, was seen several months after the attack of pneumonia. He was in a weak condition, and was expectorating quantities of fœtid purulent material. On physical examination, bronchial breathing, and a great number of small and large râles were heard towards the front of the right lower lobe in the axilla. The note obtained on percussion just below the angle of the scapula is described as having a "distinctly metallic character." The patient was operated upon, and after pus had been found by aspiration, the cavity was opened and drained. In six weeks the patient gained thirty pounds in weight, and was discharged well.

The second case was in a man, aged 59, who, after an attack of pneumonia, had lost fifty pounds in weight in four months. When first seen there was almost constant cough and expectoration of large quantities of offensive pus. On physical examination there was dulness over the greater part of the left lower lobe, and a "cracked-pot sound" could be elicited outside the lower angle of the scapula. On auscultation, the voice and breath sounds are described as having been "metallic in character." This patient, like the other, was operated upon, and the abscess cavity drained. In six weeks he had gained forty-five pounds in weight, and, later, left the hospital with the wound completely healed.

**Abscess of lung from foreign body.** Although, perhaps, the subject is more surgical than medical, it may be interesting to refer here to a case where *a foreign body which had set up an abscess in the lung* was removed by an operation which opened the abscess cavity. A boy, nine years old, when playing with a blade of grass in his mouth, drew it into the air-passages. As is usual in such cases the accident was followed by violent paroxysms of coughing, and in a few days the onset of fever suggested that the foreign body was setting up trouble within one of the lungs. At the end of about a fortnight dulness was discovered on the right side of the chest, behind and about the level of the tenth rib, and since the temperature continued to be raised, an exploratory puncture was made and pus drawn off. It was then decided to operate. An abscess in the lung was discovered; on introducing a finger into the abscess cavity

[1] *J. Am. M. Ass.*, 1902, xxxix. 739.

the foreign body was felt, and was removed with the aid of a pair of forceps. The boy made a good recovery.[1]

**Recurrent vomiting.** A subject about which one, perhaps, does not hear very much is "recurrent vomiting." Dr. Keith Shaw[2] and Dr. Larned[3] both have papers upon this affection, and record cases. The disease is one of children, and perhaps is only an exaggerated type of a not uncommon affection. Some children are subject to recurrent febrile attacks. There may be little else to be noticed beyond the ordinary accompaniments of a rise of temperature; but with many cases there is cough, and some evidence of slight bronchitis; while with others the symptoms may be abdominal, and vomiting attacks lasting for about twenty-four hours are not so very uncommon. The attacks which go by the name of "recurrent vomiting," however, are very severe, and may last two or three days. The vomiting itself is described as "uncontrollable," and is attended with "symptoms of general prostration which may be alarming." The vomiting is said also to be "at first forcible, but towards the end of the attack, when exhaustion becomes marked, is accompanied by much effort." Constipation is usually present; and frequently no action of the bowels can be obtained until the vomiting has ceased. In spite of the severity of the vomiting, after it has ceased the recovery of the patient is generally rapid, in fact is described by one writer as being "almost instantaneous." One other small point to be noticed is that as in the more common mild cases referred to above, there is usually a slight rise of temperature. Severely ill though the children are during the attacks, happily a fatal ending is rare, and only twice has such an event been recorded. The disease generally begins before the fourth year of life. The frequency of recurrence varies considerably in different cases; but they usually become less frequent as the patient grows older, and cease altogether at puberty.

Apparently little can be done to check the vomiting; but morphia may be given hypodermically, or chloral be administered by the rectum. Dr. Shaw seems to consider that chloral given in this way in one of his cases had a beneficial effect. Large rectal injections of saline solution may also be given twice a day in order to allay thirst and keep up the secretion of urine.

The etiology of the disease has been discussed by several writers, and the majority appear to consider it either due to a toxin absorbed from the intestine, or to some form of auto-intoxication. No less than four observers seem to have noticed the odour of acetone in the breath, and three of them to have found it also in the urine. The acetone is probably of slight

[1] Bechmann, *Deutsche Ztschr. f. Chir.*, vol. lxiv., No. 1, p. 3; quoted in *La Semaine méd.*, Feb. 4, 1903, p. 38.
[2] *Arch. Pediat.*, 1902, xix. 824. [3] *Am. Med.*, 1902, iv. 258

significance in itself, but is said to be always accompanied by true pathological toxins.

Dr. Larned says that the diagnosis must be easy in most cases should the medical man be aware of the existence of the disease; but remarks that he expresses this opinion "feelingly," because two years passed before he had diagnosed his first case. To anyone who has not noticed a description of cases of "recurrent vomiting," the onset of meningitis or of acute intestinal obstruction would be considered probable, at least in the first attack.

It may be mentioned that in a case seen from time to time about six years ago at the Children's Hospital, it occurred to me whether the existence of pancreatic calculi was possible. In that case, however, there was, what appears to be uncommon in these cases, epigastric pain. This absence of marked abdominal pain is important; and Dr. Shaw thinks it a point to be remembered when the question of the possibility of the presence of intestinal obstruction arises.

THEODORE FISHER.

